# Ten simple rules for getting started with statistics in graduate school

**DOI:** 10.1371/journal.pcbi.1010033

**Published:** 2022-04-21

**Authors:** Rachel A. Zitomer, Jessica Karr, Mark Kerstens, Lindsey Perry, Kayla Ruth, Lindsay Adrean, Suzanne Austin, Jamie Cornelius, Jonathan Dachenhaus, Jonathan Dinkins, Alan Harrington, Hankyu Kim, Terrah Owens, Claire Revekant, Vanessa Schroeder, Chelsea Sink, Jonathon J. Valente, Ethan Woodis, James W. Rivers

**Affiliations:** 1 Department of Forest Ecosystems and Society, Oregon State University, Corvallis, Oregon, United States of America; 2 Department of Integrative Biology, Oregon State University, Corvallis, Oregon, United States of America; 3 Department of Forest Engineering, Resources, and Management, Oregon State University, Corvallis, Oregon, United States of America; 4 Department of Animal and Rangeland Sciences, Oregon State University, Corvallis, Oregon, United States of America; 5 Department of Fisheries, Wildlife, and Conservation Sciences, Oregon State University, Corvallis, Oregon, United States of America; 6 Smithsonian Conservation Biology Institute, Migratory Bird Center, National Zoological Park, Washington, DC, United States of America

## Introduction

Graduate school is often a time of enormous professional growth, and for many students, it is the first time they receive advanced training in statistics. Despite the foundational importance of statistics in many fields, learning proper statistical approaches can be challenging for beginning graduate students, especially for those that lack prior experience in this subject area. This can occur for several reasons but is often related to competing interests for students’ limited time at the start of a graduate program. New graduate students are typically working with a new advisor, taking on new research, and/or helping to instruct a class for the first time, in addition to taking advanced courses, applying for grants and fellowships, and settling into their new professional setting. Graduate school also marks a transition to self-directed learning that may diverge strongly from undergraduate degree programs, which can also bring new challenges. In short, the start of graduate school can be a demanding time for students with much to learn in a relatively short time period.

New graduate students often begin their program by taking an overview course in statistics such as basic statistical methods, experimental design, or linear modeling. However, in the collective experience of our group—which includes graduate students, research assistants, postdoctoral researchers, and university professors in the field of natural resources—we have found that learning to apply statistical analyses correctly requires course-based learning about specific analysis methods, as well as a broader understanding of the philosophy of applying statistical approaches in research. We have found that the latter topic, on which we focus here, often gets less attention in graduate courses because it does not fit neatly into any single course. Nevertheless, topics related to this theme regularly arise throughout graduate school and beyond and thus highlight its importance for learning how to design, undertake, and complete high-quality research. Although there have been several excellent reviews in the 10 simple rules series on topics that are linked to the broader points we raise here, such as guidelines for using statistics [1], managing data [2], and learning to program [3], none have been focused on a discussion of broad conceptual topics that relate to applying statistical approaches in research with new graduate students as a primary audience. Therefore, we offer what we view as 10 important rules for new students to reflect on as they begin to build their statistical skillsets in graduate school. It is important to note that some of the rules we offer cannot be met in all circumstances; thus, we view them as rules to which graduate students should aspire, recognizing that, at times, they may be bent in the name of pragmatism. Finally, although the primary audience for the rules we provide are new graduate students, we note that the principles we raise are broadly applicable to researchers in a wide range of disciplines. Indeed, we posit that some advisors of new graduate students might themselves benefit from a periodic review of these rules!

### Rule 1: Start with a research question before data collection

New graduate students often feel pressure to get started with their research as soon as possible and that includes collecting data toward a thesis or dissertation. Despite this, it is critical to start the process by formulating a research question well before data collection begins [4]. Extensive reading of the primary literature, as well as recent review papers and edited volumes, is key to learning about the scope of work that has—and has not—been conducted in a particular field, which helps to hone and focus research questions [4]. Although it may seem more efficient to collect data while developing research questions, doing so is suboptimal and should be avoided for several reasons. First, it reduces the amount of time available to undertake a literature review expansive enough to identify the key knowledge gap(s) that could be the focus of a graduate project. Second, collecting data prior to finalizing a research question makes it more likely that key variables that need to be measured remain unidentified, at least at the start. Finally, collecting and analyzing data while developing hypotheses can lead to hypothesizing after the results are known, also known as HARKing [5]. Although it may be acceptable to revise the original hypotheses prior to data analysis, it is problematic if hypotheses are changed after data analysis is conducted and then presented as if they were developed prior to data collection [5]. Importantly, HARKing goes against the hypotheticodeductive premise of conducting science [6] in which a researcher generates a falsifiable hypothesis and then collects data to test it because, by definition, a hypothesis that has been created based on the results of an analysis cannot be falsified by the same analysis. Additional pitfalls of HARKing include a reduction in the search for alternative explanations for phenomena and strong potential for hindsight confirmation bias [5]. These issues can limit replicability and should be avoided by ensuring that a priori hypotheses are tested and that any speculation that is undertaken after results are known is clearly stated as such. Preregistration of research, wherein a research plan is deposited in a public repository before a project is undertaken [7], is a useful way of ensuring that hypotheses and analytical approaches are determined prior to data analysis, rather than in response to it. This practice increases transparency in the research process and is becoming increasingly common in many scientific fields [8].

It is worth noting that some graduate students may work on a research topic that has already been selected by their research advisor, join a project that is already underway, and/or receive a dataset that has already been collected. In such cases, they may not be starting from “square one” in the research process. Nevertheless, it is still important to have a thorough understanding of the background literature and the research question(s) that motivated data collection. Thus, rather than assuming that such an “inherited” study lacks room for improvement, we recommend that students critically examine the choices made in data collection, understand their strengths and weaknesses, and independently assess whether the resulting data are sufficient to address the research question(s) being examined.

### Rule 2: Understand how manipulative experiments differ from descriptive studies

Studies come in many forms, with descriptive studies and manipulative experiments being among the most common. Of these, the randomized manipulative experiment is the gold standard in terms of inference [9], and in its simplest form, it is a representative group of experimental units (e.g., individuals, study sites) from a population that is randomly assigned to either a treatment or a control group. Units in the treatment group are subjected to an experimental manipulation, whereas those in the control group are not, and the average response across experimental units in the 2 groups is then compared. Because other factors are held constant, differences detected in the treatment group relative to the control group can be attributed to the experimental manipulation. In contrast, observational studies are those that describe relationships as they occur, without manipulation by a scientist, who in this case serves as a passive observer and not as a manipulator of the system under study. The differences between these 2 types of studies may seem subtle, but they have enormous importance for inference, and the strength of conclusions that can be drawn. Most importantly, randomized experiments can assign causation to the factor that is manipulated by a researcher, whereas descriptive studies are limited to providing correlative evidence of relationships [10]. It is critical to note that despite these differences, descriptive studies play an important role in generating knowledge; they are, in fact, the foundation for much of science as many systems do not allow for manipulation, especially across large scales [11]. However, their limitations must be understood relative to manipulative experiments so that proper inference is made (see Rule 3).

### Rule 3: Understand the limits of inference

Inference in research involves drawing conclusions about a population based on observations of a sample of that population using inductive reasoning. The validity of inductive reasoning is dependent on the premise that the sample is representative of the population of interest [12]. The set of situations to which the conclusions of a study can be generalized is known as its scope of inference [13]. The ideal for most researchers is to apply the results of research as broadly as possible. However, the reasonable scope of inference for a research project is typically constrained by the realities of data collection. Thus, defining the scope of inference for a study involves thinking carefully about the chosen sampling approach, potential sources of bias, and acknowledging how they limit the generalizability of a study. Likewise, adopting an approach that is too narrow when defining the scope of inference for a study can also be limiting. Some graduate students may focus so intently on the specifics of their study that they fail to consider the larger range of conditions that it might represent. In some cases, the scope of inference for a study may be broadened considerably if there is evidence that the conditions in which sampling occur are typical of a larger geographic area or timeframe. In other cases, studies with a relatively narrow scope of inference might fill a gap in the existing body of research on their subject that allows more generalizable conclusions to be drawn. Thus, defining the scope of inference for a study requires a striking a balance where the scope is narrow enough that findings are appropriately applicable, yet broad enough to provide knowledge that extends beyond a single study system. Getting feedback from colleagues and peers about what is a reasonable scope of inference for a particular study is often a useful exercise (see Rule 10).

### Rule 4: Start learning a statistical programming language early

Although some statistical programs are “canned” and do not require extensive knowledge of programming to perform statistical analysis, our experience has found that graduate students who learn a programming language early in their program become more proficient with data analysis throughout graduate school and beyond. Although it does take time, learning a programming language helps to solidify statistical concepts because it requires creating all of the steps needed to undertake an analysis, in marked contrast to statistical programs operated through a graphical user interface. In addition, learning how to program in a commonly used language increases efficiency and accessibility of analyses [14], especially when well-annotated scripts are created that can save time when code is reused in subsequent analyses. Using a statistical programming language can also increase reproducibility as scripts not only enable data manipulation and statistical testing but also record the nature and sequence of these steps [15]. Archiving all versions of scripts and datasets—including raw data—is a straightforward and efficient way to track changes made throughout the analysis process [16]. It is important to note that programming scripts should not be the only mechanism for ensuring reproducibility [16], but they are particularly useful for keeping a record of each step in the analysis so that important details are not lost.

We suggest that new students consult literature and talk with others in their field to find out which programming languages are commonly used and to understand their strengths and weaknesses with different types of data or analytical approaches before choosing the language(s) on which to focus [3]. For example, one programming language that is free and used widely in the natural resource sciences [17] is the R statistical environment (https://www.r-project.org). Because it is an open-source language, there are many packages that are created for specific analyses and freely available to researchers. There are also extensive tutorials and a rich online public support community that can provide relevant code and troubleshoot errors that may arise. Regardless of whether one uses R or another programming language (e.g., SAS, Python), the work that new graduate students put into learning programming early in graduate school will pay dividends later as they move beyond graduate school and into the next phase of their career.

### Rule 5: Set up databases in a tidy format before data collection

Graduate students vary in how they obtain the data they use for their graduate research. Although some students are fortunate to receive a well-curated database in its final form, most end up spending a large amount of time wrangling their data. Indeed, it is estimated that up to 80% of data analysis time is spent cleaning and preparing data prior to conducting any statistical analyses [18]. Although different types of data analyses often require different data formats, there are some general rules to keep in mind when developing a database, and they should be implemented prior to data collection. First, standardize data entry through the establishment of metadata that, at a minimum, describes the actual names of the variables found within a database (e.g., “body_mass”), provides a definition for each variable (e.g., “body mass measurement taken with an electronic scale to ± 0.01 g”), and reports possible entries for each variable (e.g., “positive continuous values”). Including this information is critical so that anyone in possession of the data can clearly understand individual variables and how they were defined. Additionally, structure databases so that each variable forms a column, each observation forms a row, and each type of observational unit forms a table [19]. This structure—referred to as “tidy data”—is an ideal format for storing data that facilitates statistical analyses. Wickham [19] extolls the many virtues of this approach, and his paper on tidy data is a must-read for any new graduate student. Storing data in a tidy format also make it easier to reproduce results, when returning to previously used datasets and when sharing data with other researchers, both of which are critical for building scientific knowledge [1].

### Rule 6: Understand the form of data

Once data are in hand (note that data is a plural term and datum is singular), whether they are collected directly or obtained indirectly, it is essential to have a good understanding of those data and how they should be analyzed. Introductory statistics courses typically focus on general linear models (e.g., *t* tests, linear regression, and analysis of variance) that assume that measured responses are normally and independently distributed around the modeled mean. The normality assumption in particular can be a source of great anxiety for many new graduate students when they discover their residuals do not follow such a form, often leading to arcane data transformations or even abandonment of certain data that have been collected. Thus, it is critical to recognize that techniques are now widely available for analyzing data with a variety of forms, including those that are binary (e.g., logistic regression and Fisher exact test) or are based on counts (e.g., Poisson and negative binomial regression) or proportions (e.g., Dirichlet regression). Of course, each of these approaches comes with its own assumptions, and, therefore, it is critical to ensure that they are met regardless of the method chosen. In some cases, nonparametric approaches, which tend to have fewer assumptions but also have lower statistical power, may be reasonable alternatives. Regardless, being able to understand the form of the data one has and identify an appropriate underlying distribution when evaluating statistical hypotheses with those data are critically important skills for new graduate students given the many analytical options available today.

In many sampling situations, the assumption of independence that underlies general linear models, as well as many other analytical techniques, may also be violated. Sometimes, nonindependence is purposeful; for example, a researcher may be interested in how herbicide exposure affects salamander body weight over time. In this case, multiple body weight measurements on the same salamander are not independent of one another, yet they are required to answer the research question. In other cases, nonindependence can be a side effect of sampling constraints, such as when samples collected closer together in space or time are more likely to have similar values due to environmental homogeneity at local scales. Several techniques have been developed to account for these sources of nonindependence, including mixed model analysis [20] and diverse methods for handling spatial and temporal autocorrelation [21,22]. New students should be aware that nonindependence in data must be recognized and accounted for, and failing to do so can lead to pseudoreplication and other issues related to data interpretation [23].

### Rule 7: Understand what a *p*-value is and what it is not

*P*-values have been frequently overemphasized and misinterpreted in the scientific literature [24]. Technically defined, a *p*-value is the probability of obtaining an observed dataset if the null statistical hypothesis were true. Typically, a null hypothesis postulates the absence of an effect; that is, that there is no difference between groups being compared or no association between variables being evaluated [25]. A very small *p*-value therefore suggests that it is unlikely one would have obtained the observed results if there truly was no effect. *p*-Values are traditionally compared to a threshold value, alpha (α), which is defined a priori and serves to delineate the acceptable probability of mistakenly rejecting the null hypothesis when it is true [26]. This approach frequently results in interpretation of an observed effect as “significant” or “nonsignificant” if the *p*-value is smaller or larger than α, respectively [27].

It is critically important for new graduate students to recognize that a *p*-value is a function of the magnitude of an effect, the variability in a response, and the sample size of a dataset. Because of this, even trivial effects may be deemed statistically “significant” when sample sizes are sufficiently large [25]. Conversely, strong effects may not be reflected in the outcome of statistical tests when sample sizes are small or when variability in a response is large. For example, 2 studies could estimate similar effect sizes yet differ in the precision of their estimates—for example, if one study has a small error estimate and the error estimate of the other study is large—leading one to conclude the first study detected a statistically “significant” effect, whereas the second study was found to have no effect at all [26]. In reality, there is no conflict between these 2 hypothetical studies regarding the observed effect, yet adopting the use of statistical “significance” when describing their findings suggests that such a conflict exists, which can lead to erroneous conclusions [26]. Therefore, new graduate students should take care to appreciate that *p*-values should not be taken as an indicator of whether the effect of a particular variable is strong or has importance to the system under study. Instead, *p*-values are merely a means of quantifying how certain one can be that there is no effect, given the type and quantity of data that have been collected. The American Statistical Association’s statement on *p*-values provides excellent guidance on the use and interpretation of *p*-values and clarification of common mistakes and is as recommended reading for all new graduate students [27].

### Rule 8: Learn how statistical power can influence results

In any study, there are constraints on the amount of data that can be collected. Therefore, it is important to understand the role of statistical power and sample size in influencing results, as well as the scope of inference [28] (see Rule 3). Statistical power is the probability of correctly rejecting a statistical null hypothesis that is false, and this probability increases as both sample size and effect size increase. Thus, it may be possible to detect differences between 2 populations even with small sample sizes if effect sizes are large, whereas large sample sizes might lead to detecting very small statistical differences [29]. Given these considerations, it is essential to turn a critical eye when examining results to the extent to which methodological effects (e.g., sample size) may be driving the results relative to the strength of the response in the system (i.e., effect size). Conversely, when sample sizes are small and effect sizes are either small or data are highly variable, statistical power to detect differences is low [25]. In such instances, it is important to recognize the old adage that “the absence of evidence is not evidence of absence” and that an effect may be present even though low statistical power does not allow researchers to detect it within a study. Furthermore, reporting such negative results should also reflect this possibility; for example, it is preferable to state that no differences were detected in a study rather than stating that no differences were present. Although retrospective power analysis is generally viewed unfavorably, power analysis may, in some instances, be useful when applied a priori to determine what sample size is necessary to detect the minimum effect size of interest given a specified expected variance [28].

### Rule 9: Appreciate the importance of effect size

As noted above in Rule 7, statistical null hypotheses posit that there is no difference between groups being compared or no association between variables being studied [25]. In practice, however, there is almost always some expected difference in measures taken between any 2 populations, however small, simply due to sampling effects and random variability [30]. Thus, the purpose of performing statistical analysis is not usually to determine whether 2 groups are absolutely the same, but rather whether the difference between them (i.e., the effect size) is meaningful within the context of the study system.

The effect size is the magnitude of a relationship between variables or a difference among groups [31], and it is typically presented with confidence intervals that represent the degree of uncertainty around the estimate [24]. In most cases, estimates of effect size are more informative than *p*-values because they provide context for the magnitude and direction of an effect and therefore should be emphasized when evaluating and reporting results [30]. For example, stating that a species of interest was “3.2 × (95% CI: [1.3, 4.6]) more likely to use a treatment site than a control site” is more informative than stating that “there was a significant effect (*p* < 0.01) of treatment on site use.” The former approach highlights the magnitude of the effect, which, in turn, may help resource managers evaluate whether the biological response is worth the cost of implementing similar treatments in the future. In other words, interpreting the results in the context of the system under study is what really matters, without undue focus on statistical outcomes.

### Rule 10: Don’t fly solo

If you are a new graduate student who feels a bit overwhelmed by the previous rules outlined here, this last rule is especially for you. Although it may seem like you are embarking on a singular odyssey by yourself, graduate school is really a time to develop connections with new peers, colleagues, and mentors. As such, it is important to develop and make use of a new professional support network throughout graduate school, including in the exploration of statistical analysis techniques and theory. We have found that statistics can be especially challenging for new graduate students and that learning new techniques and programming languages is best facilitated by collaboration and conversation with others. In addition to the benefits that derive from working with other students, there are often other outlets that can provide help when learning statistics. For example, some universities have statistical consultants that provide tailored one-on-one help that can provide input and assistance on myriad topics. With regard to working with consultants, one oft-repeated piece of advice is to seek help from statisticians before data collection begins, as that is the phase of the research process in which they can best help to remedy problems that may be present in a study; when the time has come for data analysis, it is often too late. More broadly, there is a wealth of online tutorials on statistical methods and programming languages, as well as free online courses, which combine statistics and programming and extensive online help communities for many programming languages. In short, make sure you tap into the resources that are available so that when—not if—you get stuck you will have a support system that can help get you back on track.

## Concluding thoughts

Graduate school is an exciting time, but it can also be stressful due to its finite nature. In many fields, developing an understanding of statistics is a key part of the professional growth of graduate students, and it takes many forms: classes, workshops, self-study, and more. Given its central importance, and the expanding body of statistical science, our collective experience has found that researchers must continue to learn about statistical methods throughout their career, and, thus, developing a solid base early in one’s graduate program is essential for continued growth and development. It is therefore our expectation that these rules, when taken together, will help make that process a little less daunting for new students as they develop a deeper understanding of the statistical methods they will use during graduate school and throughout the rest of their careers.
